# Habituation to Livestock Trailer and Its Influence on Stress Responses during Transportation in Goats

**DOI:** 10.3390/ani13071191

**Published:** 2023-03-29

**Authors:** Govind Kannan, Phaneendra Batchu, Aditya Naldurtiker, Gregory S. Dykes, Priyanka Gurrapu, Brou Kouakou, Thomas H. Terrill, George W. McCommon

**Affiliations:** Agricultural Research Station, Fort Valley State University, Fort Valley, GA 31030, USA

**Keywords:** catecholamines, goats, habituation to livestock trailer, stress, transportation

## Abstract

**Simple Summary:**

Long-duration transportation of goats can cause significant changes in their behavior and physiology that could compromise their welfare. This study was conducted to determine if repeated exposure to livestock trailers can be beneficial in reducing stress responses due to prolonged transportation in goats. A total of 168 uncastrated male Spanish goats were divided into groups and maintained on two identical paddocks. One group was fed a concentrate supplement inside two livestock trailers for 4 weeks, and the other group was fed the same diet in the feeding area with no trailers. After the habituation period, the goats were transported for 10 h in four replicates, and blood samples were collected every two hours to evaluate stress responses. Phenylethylamine and 5-methoxytryptamine concentrations were significantly higher in the group habituated to trailers compared to the group not habituated to trailers. Dopamine, 5-methoxytryptamine, and metanephrine concentrations were significantly influenced by transportation time. The blood biogenic amine concentrations indicate that the habituation of goats to livestock trailers may be beneficial during transportation for long periods.

**Abstract:**

This experiment was conducted to determine the effects of habituation to livestock trailers on stress responses in goats transported for long periods. Intact male Spanish goats (12-month old; BW = 31.6 ± 0.34 kg; N = 168) were separated into two treatment (TRT) groups and maintained on two different paddocks. Concentrate supplement was fed to one group inside two livestock trailers (5.0 × 2.3 m each; habituated group, H), while the other group received the concentrate supplement, but not inside the trailers (non-habituated, NH). After 4 weeks of habituation period, goats were subjected to a 10-h transportation stress in four replicates (*n* = 21 goats/replicate/TRT). Blood samples were collected by a trained individual by jugular venipuncture into vacutainer tubes before loading (Preload), 20 min after loading (0 h), and at 2-h intervals thereafter (Time) for analysis of stress responses. There was a tendency for a TRT effect (*p* < 0.1) on tyramine and metanephrine concentrations. Phenylethylamine and 5-methoxytryptamine concentrations were significantly greater (*p* < 0.05) in the H group compared to the NH group. Both dopamine and 5-methoxytryptamine concentrations decreased (*p* < 0.05) with transportation time; however, TRT × Time interaction effects were not significant. Habituation to trailers may be beneficial in mood and energy stabilization in goats during long-distance transportation.

## 1. Introduction

Transportation is one of the most stressful events that goats raised for meat production undergo and the intensity of stress experienced can depend on the distance, duration, road condition, hauling method, and handling practice that vary in different parts of the world [1]. Although meat goats are typically transported 2–3 h from farm to processing plants in the US [1], personal communications with farmers and ranchers show that when goats are sold from one farm to another, they are often transported long distances. For example, meat goats from western Texas are shipped to different states on the east coast of the US, journeys that can last up to 18 h. Transportation for prolonged durations can cause significant physiological changes in goats that can negatively affect their well-being [1]. It is imperative to find management methods suitable for industry application that will attenuate stress responses due to extended transportation in goats.

Increases in cortisol concentrations and creatine kinase (CK) activities were observed in goats transported for 2 ½ h [1]. Transportation stress has been reported to increase concentrations of epinephrine, norepinephrine, and dopamine in small ruminants [2]. A recent study [3] has suggested that under practical conditions, plasma metanephrine and normetanephrine concentrations could be accurate indicators of stress in goats as the values were significantly correlated with plasma cortisol concentrations. Metanephrine and normetanephrine are breakdown products of the catecholamines, epinephrine and norepinephrine, respectively. The use of catecholamine breakdown products and other biogenic amines as potential indicators of stress under different commercial handling situations in goats has not been studied adequately. These hormones and neurotransmitters, which play a crucial role in an animal’s response to stress, are produced via both brain and peripheral systems. The brain catecholaminergic systems include the noradrenergic system, adrenergic system, and dopaminergic system, and L-DOPA neurons [4]. Recent reports have shown there are three different peripheral catecholaminergic systems [5]. These include the sympatho-adrenomedullary system, sympatho-neural system, and the DOPA-dopamine autocrine/paracrine system. As activation of these systems in response to stress invariably results in an increase in circulating levels of catecholamines, these hormones and their derivatives prove to be good indicators of stress in goats [3]. In addition, some biogenic amines have been reported to influence mood and energy stabilization and antioxidant status in mammals [6,7] and thus could be explored as potential indicators of these statuses in goats.

Appropriate habituation techniques can help animals get acclimated to a novel environment, such as a transport trailer, and reduce the stress related to that technique or environment [8]. Learning, which can be defined as a somewhat enduring change in behavior resulting from familiarity, can be broadly classified as associative and non-associative learning [9]. Habituation, which can fall under non-associated learning, refers to the attenuation of a response to a repeated same stimulus that likely elicited a stronger response when exposed the first time [10]. Animal scientists have studied numerous habituation techniques to reduce stress responses to routine handling and management procedures in food animals such as repeated gentle handling and passage through the working chute with varying results [11,12]. Although studies have shown that repeated exposure to transportation can reduce stress responses in animals [13], it is not practicable under commercial livestock production conditions. We propose that acclimating goats to livestock trailers for a few weeks before impending long-duration transportation could be a viable alternative to repeated exposure to transportation.

Based on our research, there is no information available on the effects of habituation of goats to livestock trailers on their stress responses, particularly changes in catecholamine profile, during transportation. We believe this study will provide a valuable practical solution to reducing handling stress in goats during transportation. This experiment was conducted to determine the effects of habituation to livestock trailers on behavior, plasma cortisol concentrations, creatine kinase activities, differential leukocyte counts, and catecholamine profile in goats transported for long periods.

## 2. Materials and Methods

### 2.1. Animals

The animal care protocol for this study was reviewed by the FVSU Agricultural and Laboratory Animal Care and Use Committee following the ADSA-ASAS-PSA Guide for Care and Use of Agricultural Animals in Research and Teaching [14] and approved (Approval # S-R-02-2021). A total of 168 intact male Spanish goats were separated into two treatment (TRT) groups and maintained on two different paddocks, with 84 goats in each paddock. The goats had not been subjected to any prior handling except for deworming and weighing that was done 4 weeks before the beginning of the experiment. Although flight distance was not measured, all 168 goats were treated similarly before the study and were not easily approachable. A concentrate pellet supplement was fed to one group inside two livestock trailers (5.3 × 2.3 m each; habituated group, H), while the other group received the concentrate supplement (18% crude protein, 4% crude fat, 15% crude fiber) in the feeding area of the paddock with no trailers (non-habituated, NH). For the H goats, two linear feeders were placed in each of the two trailers used for habituating goats. Since not all 84 goats could feed in the two trailers at the same time, the goats were allowed access to the trailers in three batches (28 goats in each batch) with 14 goats in each trailer for 50 ± 10 min every day. This was approximately the time it took for all goats to consume the feed, stop eating, and move away from the feeders. The feeder space available for the 14 goats in each trailer was adequate to prevent any agonistic encounters during the 50-min feeding time every day. The total quantity of concentrate pellet feed given was 68 kg for 84 goats in the H paddock and the same quantity for 84 goats in the NH paddock. The feeding areas in the two paddocks were at opposite corners such that the NH goats could not see the trailers. Since the goal of this study was to find a practicable method to reduce stress during long-distance transportation, a 4-week habituation period was deemed long enough for goats to become acclimated to the trailer. This was based on the observation that the H goats readily entered the trailers after the first few days and sometimes laid down inside the trailers after feeding.

The term “habituation” was used for ease, although the conditioning technique involved both associative and non-associative learning. When an animal learns a new response by associating two or more events (ex. operant conditioning), it can be categorized as associative learning. Non-associative learning is referred to the change in behavior of an animal in response to repeated exposure to an event or stimulus (ex. habituation) [9]. In our experiment, since the treatment applied did not clearly fall into either category, but rather had both aspects to it, we used the term “habituation.” After 4 weeks of habituation period during the months of March–April, goats were subjected to 10-h transportation stress in 4 replicates (*n* = 21 goats/replicate/TRT; floor space 0.29 m^2^/goat for a total of 42 goats on the trailer per replicate). The time taken for herding the goats in each treatment group into smaller pens before loading and the time taken for the loading process itself were recorded on each day. The average temperatures on the days of transportation ranged from 17.8 to 23.1 °C, and the average relative humidity ranged from 68.0 to 79.0%. One of the trailers used for habituation was also used for transportation of goats. The all-aluminum trailer (Featherlite, Model 8107, Featherlite Trailers, Cresco, IA, USA) had a skid-resistant extruded aluminum floor, a curb-side escape door, and one full-swing center gate with a slam latch. Using the center gate, the trailer was divided into two compartments, one for each TRT, which was alternated between compartments on each day. A ramp was not used for loading and unloading goats since the trailer floor was only 32 cm from the ground level. Blood samples were collected from both TRT groups before loading (Preload), 20 min after loading (0 h), and at 2, 4, 6, 8, and 10 h of transportation (Time) for analysis of plasma cortisol concentrations, CK activities, leukocyte counts, and catecholamine profile. A split-plot design was adopted for this experiment, in which TRT was the whole plot and Time was the subplot.

### 2.2. Behavioral Observations

Two observers, one for each treatment compartment in the trailer, conducted behavioral observations during the 20-min period between completion of loading and 0-h blood sampling (20 min after loading) to determine if there were TRT or Time differences in the demeanor of goats after loading. Each observer stepped onto the 30-cm-wide platform on the side of the trailer and observed the behaviors of goats through the window. The observers stayed still and quiet throughout the 20-min period to not influence the behaviors. The observers were not blinded to the treatments but alternated between treatments in each replicate. The number of goats performing behaviors such as standing, moving, agonistic, climbing, urination, and defecation, was recorded. Standing behavior was recorded when a goat stood still without taking a step in any direction. Moving behavior was recorded when an animal moved by taking at least a step forward or backward. Agonistic behavior was recorded when the goats engaged in agonistic encounters, and climbing behavior was recorded when a goat lifted its forelimbs from the floor in an attempt to escape the situation. The observer used a scan sampling method every minute to record the number of goats performing each behavior. Standing and moving behaviors were mutually exclusive. To determine if the behaviors changed with time within the 20-min period, the data were grouped into four 5-min segments. Urination and defecation behaviors were not included in the analysis since these behaviors could not be recorded accurately due to the body orientation of some animals in relation to observers’ positions.

### 2.3. Blood Sampling

Blood samples were collected by a skilled individual by jugular venipuncture into two different vacutainer tubes. The blood tubes for separation of plasma and those for leukocyte counts contained K_2_EDTA and EDTA (K3) coating, respectively. The tubes were kept on ice until separation of plasma. The individual who collected blood samples was so proficient that there was no time lapse in collecting the blood sample after a goat was caught (<30 s). To avoid repetitive unloading and loading of goats, the truck was stopped for 10 min at 2, 4, 6, and 8 h sampling periods, and blood samplings were conducted inside the trailer. All possible efforts were made not to agitate the goats, and only two individuals entered the trailer for blood sampling, one animal handler, and one blood sampler. At each time period, a different set of goats were sampled, as color markers were used to mark the horns of each goat after blood sampling to avoid being sampled again. For separation of plasma, the tubes were then centrifuged at 1000× *g* for 20 min. Plasma samples were pipetted into screw-cap vials and stored at −80 °C until analysis.

### 2.4. Cortisol

A commercial ELISA kit (Cortisol ELISA Kit, Abnova, Taipei, Taiwan) was used to analyze plasma cortisol concentrations according to the manufacturer’s instructions. The procedure involved several steps: (i) 25 µL of goat plasma samples was added to coat the 96-well microplates, (ii) 100 µL of cortisol enzyme conjugate solution was added, (iii) incubation at 37 °C, (iv) the plates were washed four times, (v) 100 µL of color reagent (3,3′,5,5′ -tetramethylbenzidine, TMB) was added to each well, and then (vi) 50 µL of the stop solution was added to stop the reaction. A Synergy HTX Microplate Reader (Bio-Tek, Winooski, VT, USA) was used to measure the absorbance at 450 nm, and finally, the cortisol concentrations were determined against a standard curve created using the standards provided by the manufacturer.

### 2.5. Creatine Kinase

A commercial Creatine Kinase Assay Kit (Abnova Corporation, Tapei, Taiwan) was used to determine plasma CK concentrations. This kit is based on the principle that phosphocreatine and adenosine diphosphate are converted by CK to creatine and adenosine triphosphate (ATP). The ATP is used to phosphorylate glucose by hexokinase to generate glucose-6-phosphate, which is then oxidized by nicotinamide adenine dinucleotide phosphate in the presence of glucose-6-phosphate dehydrogenase. The nicotinamide adenine dinucleotide produced is proportional to the CK activity in the analyzed plasma sample.

### 2.6. Differential Leukocyte Counts

Blood samples collected separately in 3 mL vacutainer tubes coated with EDTA (K3) were used for differential leukocyte counts. A VetScan HM5 Haemotology Analyzer (Abaxis, Union City, CA, USA) was used to determine neutrophil (N), lymphocyte (L), monocyte, and eosinophil counts according to the manufacturer’s instructions.

### 2.7. Catecholamines and Other Biogenic Amines

Plasma samples (*n* = 21 goats/replicate/TRT; total 168 samples) were shipped on dry ice to the Metabolomics Innovation Center (TMIC) at the University of Alberta, Edmonton, Canada, for catecholamine profiling using the method described by Zheng et al. [15]. A combined direct injection mass spectrometry with a reverse-phase LC–MS/MS custom assay using an ABSciex 4000 Qtrap tandem mass spectrometry instrument (Applied Biosystems/MDS Analytical Technologies, Foster City, CA, USA) with an Agilent 1260 series UHPLC system (Agilent Technologies, Palo Alto, CA, USA) was used for analysis of catecholamines and other biogenic amines. The data were analyzed using Analyst 1.6.2.

### 2.8. Statistical Analysis

Behavior data were analyzed using Friedman’s Two-Way ANOVA by Ranks Test (non-parametric). Blood data were analyzed using MIXED procedures in SAS (Version 9.4, SAS Institute, Cary, NC, USA) with TRT, Time, and their interactions as fixed effects and animals as random effects. The data were checked for normality and homogeneity of variance using Shapiro–Wilk’s test and Levene’s test, respectively. When required, the data were transformed to log scale to meet the assumptions of ANOVA. When significant by ANOVA at *p* < 0.05, the means were separated using the LSD Test. Principal component analysis (PCA) was also performed for catecholamines and their derivatives using Metaboanalyst R (https://github.com/xia-lab/MetaboAnalystR (accessed on 16 February, 2023)). A median batch effect correction method described by Rusilowicz et al. [16] was used to remove the day (replicate) effect.

## 3. Results

### 3.1. Behavior

The behaviors of goats after loading were not affected by treatment (Table 1); however, moving and standing behaviors were significantly influenced by time (*p* < 0.01). The number of goats that moved decreased during the third 5-min segment, thereby increasing the value for standing behavior, since these two behaviors were mutually exclusive. Habituation to trailer appears to facilitate the loading process, as the goats readily went into the trailer, thereby reducing the time taken for this process. The average times taken to load the H and NH goats onto the trailer were 21 ± 5 s and 48 ± 5 s, respectively, although no statistical analysis was conducted. In addition, the time taken for herding goats into smaller confinement before loading them onto the trailer was much shorter and the process smoother with the H group (480 ± 120 s) than with NH group (1800 ± 240 s).

### 3.2. Cortisol, Creatine Kinase, and Leukocyte Counts

**Figure 1:**
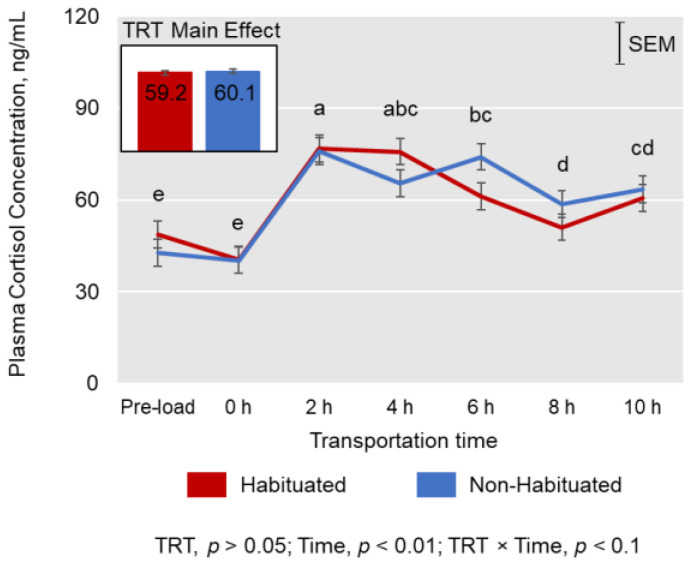
Effects of habituation treatment (TRT) and duration of transportation (Time) on plasma cortisol concentrations in Spanish goats. ^a–e^ Time main effect means (average of the two means at each time point) with different letters differ significantly by LSD test at *p* < 0.05.

**Figure 2:**
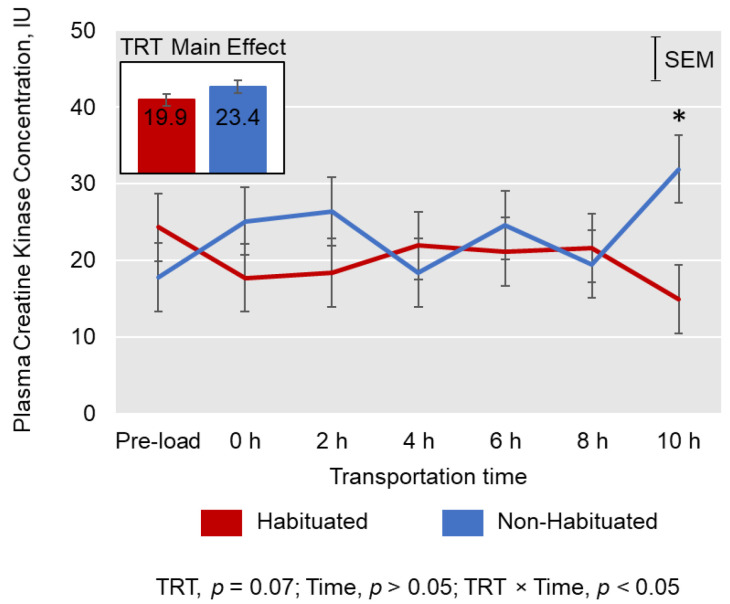
Effects of habituation treatment (TRT) and duration of transportation (Time) on plasma creatine kinase activities in Spanish goats. * TRT means at 10 h were significantly different by LSD test at *p* < 0.05.

Plasma cortisol concentrations were not affected by TRT (*p* > 0.05); however, the levels were influenced by Time (*p* < 0.01; Figure 1). Cortisol concentrations peaked at 2 h of transportation in both TRT groups and decreased thereafter. Plasma CK activities tended to be lower (*p* < 0.1) in the H group compared to the NH group, and there was a significant TRT × Time effect (*p* < 0.05; Figure 2) on CK activities, with the values increasing in the NH group and decreasing in the H group during the first two hours of transportation. Among the different types of leukocytes counted, lymphocyte (L) count decreased (*p* < 0.01) and neutrophil (N) count and N/L ratio increased (*p* < 0.01) over Time (Table 2).

### 3.3. Catecholamines and Other Biogenic Amines

**Figure 3:**
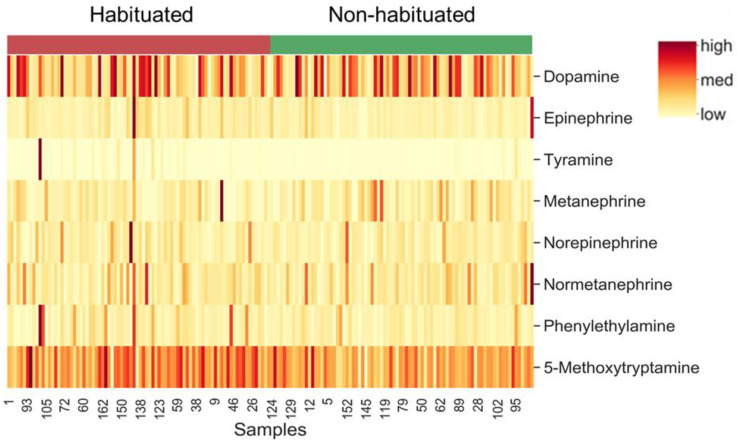
Heat map of plasma catecholamines clustered by treatment (H = Habituated; NH = Non-habituated; *p* < 0.05).

**Figure 4:**
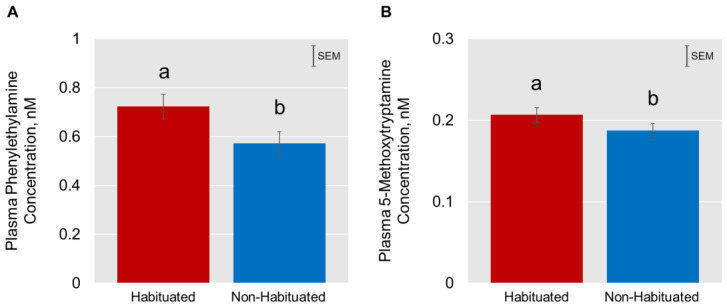
Effects of habituation treatment on plasma (**A**) phenylethylamine and (**B**) 5-methyltryptamine concentrations in goats. ^ab^ Bars with different letters differ significantly (*p* < 0.05) by LSD test.

**Figure 5:**
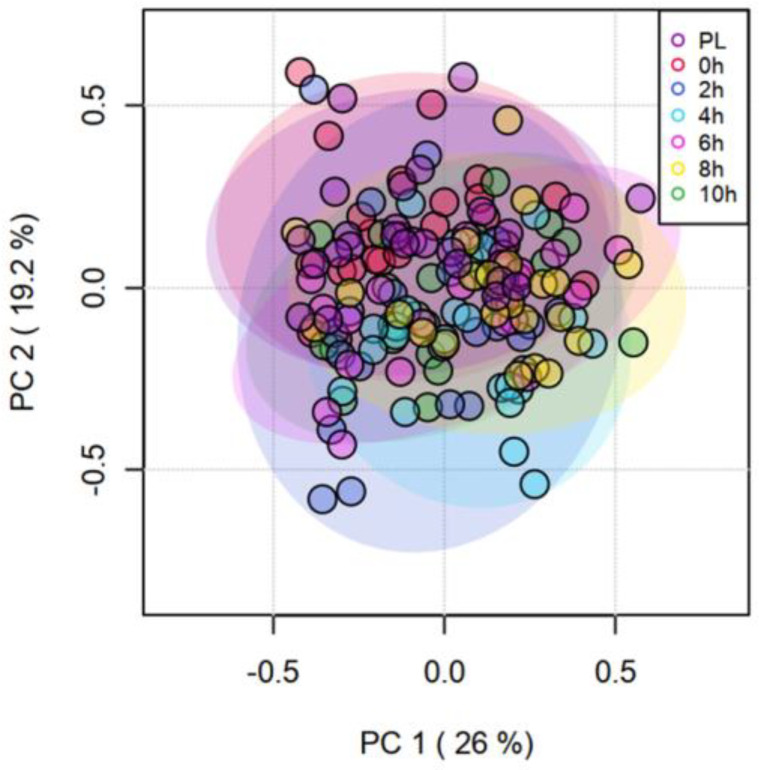
Principal Component Analysis (PCA) plot of principal components 1 and 2 of transportation time for catecholamines.

**Figure 6:**
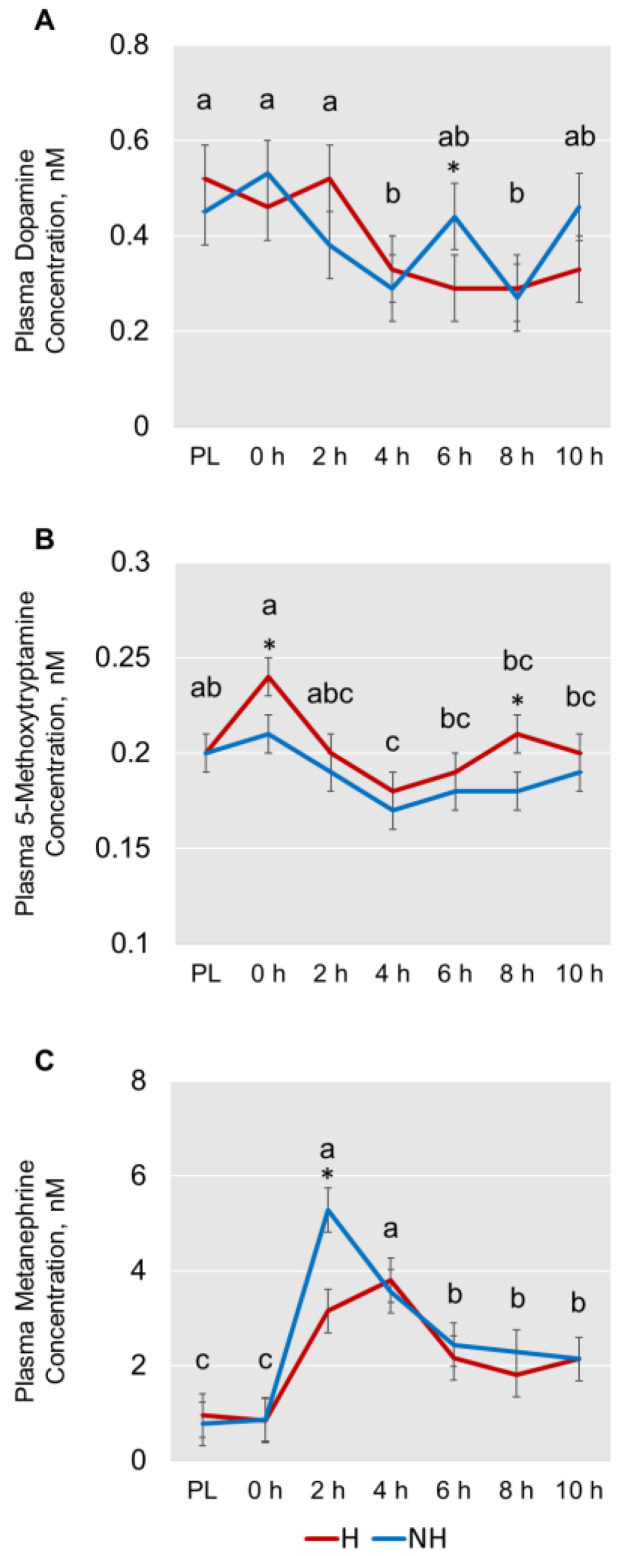
Plots of means (± SEM) showing changes in catecholamine ((**A**) dopamine; (**B**) 5-methoxytryptamine; (**C**) metanephrine) concentrations over time (PL = Preload; Time main effect, *p* < 0.05). ^a–c^ Time main effect means (average of the two means at each time point) with different letters differ significantly by LSD test at *p* < 0.05. * TRT means at the time points indicated were significantly different by LSD test at *p* < 0.05.

The effects of TRT on the concentrations of catecholamines and their derivatives are depicted using a heatmap (Figure 3) created using normalized concentration ranges (from 0 to 1) of catecholamines and biogenic amines with high, medium, and low points on the color bar corresponding to 0.95, 0.5, and 0.05, respectively. Phenylethylamine and 5-methoxytryptamine concentrations were significantly greater (*p* < 0.05) in the H group compared to the NH group (Figure 4). Habituation did not have any significant effect on dopamine, epinephrine, norepinephrine, and normetanephrine concentrations, while there was a tendency for TRT effect (*p* < 0.1) on tyramine and metanephrine concentrations. The mean (±SEM) tryramine concentrations were 3.55 ± 1.275 and 0.654 ± 1.264 nM, respectively, in H and NH groups, and the mean metanephrine concentrations were 2.121 ± 0.175 and 2.477 ± 0.175 nM in H and NH groups, respectively. Principal component analysis (PCA) plot of the time effect on the eight biogenic amines is shown in Figure 5. The PCA plot to visualize the separation of biogenic amines by Time in principal components 1 and 2 revealed that the clusters corresponding to different time periods overlapped with no clear separation. Dopamine and 5-methoxytryptamine concentrations generally decreased (*p* < 0.05) with transportation time (Figure 6A,B). The Time effect for plasma metanephrine concentrations was also significant (*p* < 0.01; Figure 6C); the levels increased and peaked at 2 h in the NH group, while the levels increased rather gradually and moderately during the first 4 h of transportation in the H group before decreasing. The TRT × Time interaction effect was not significant, however.

## 4. Discussion

### 4.1. Behavior

The standing, moving, agonistic, and climbing behaviors of goats observed for 20 min after loading were not affected by TRT. Ten minutes after loading, the number of goats that showed moving behavior decreased before increasing again during the last 5-min segment of the 20-min period of behavioral observation, although the reason for this pattern is not clear. However, the time taken to load the goats onto the trailer on the days of the experiment was shorter in the H group compared to the NH group as the H goats readily entered the trailer without hesitation. Dai et al. [13] reported that the time taken for loading donkeys habituated to transport was significantly shorter and required fewer human interventions compared to control donkeys. The authors also observed that stress-related behaviors were less frequent in the habituated donkeys compared to control donkeys. In a qualitative behavioral assessment during 90-min transportation in sheep, Wickham et al. [17] observed that transport-naïve animals were more alert, anxious, and aware, and transport-habituated sheep were more comfortable, tired, and confident. Behavioral observations were not recorded during transportation in our experiment. In order to prevent transport-related behavioral problems in horses, Houpt and Wickens [18] suggested as the first step to desensitize them to the trailer by parking the trailer in the paddock and providing feed inside it. A similar technique followed in the present experiment to condition goats to trailers involved both associative and non-associative learning, since the goats were not only desensitized for 4 weeks, which allowed sufficient time for familiarization, but were also fed inside the trailer during this period. This habituation period was long enough for them to gain assurance and be acclimated to being inside the trailer.

### 4.2. Cortisol, Creatine Kinase, and Leukocyte Counts

Habituation to the trailer did not affect plasma cortisol concentrations; however, the levels were significantly influenced by transportation time, which is in agreement with previous reports [1,2]. There are no published data on the effects of habituation to livestock trailers on stress responses in meat goats, and there have been conflicting reports on the effects of habituation to transport itself on cortisol concentrations in other livestock species. In donkeys, habituation to transport did not significantly reduce salivary cortisol concentrations compared to the controls, although the variability in cortisol concentrations was greater in non-habituated donkeys compared to the habituated donkeys [13]. In contrast, Wickham et al. [17] observed that cortisol concentrations were significantly greater when sheep were transported for 90 min for the first time (naïve event) than when they were transported for the seventh time (habituated event) within an 8-day period. The preload cortisol concentrations observed in the present study are higher than those recorded in previous studies, which may indicate that both H and NH goats experienced higher stress levels under the experimental conditions described. However, the increase in cortisol concentrations during transportation is consistent with the values reported in previous studies in goats, although when transported under extreme weather conditions, cortisol concentrations can reach as high as 130 ng/mL [19]. The baseline cortisol concentrations reported in Spanish goats in previous studies are in the range of 10–15 ng/mL [1,20,21,22]. Glucocorticoids serve as good indicators of the response of goats to any change in their environment [2,23]. However, Broom [24] urged caution in interpreting cortisol data, since some poor welfare situations, such as shelter needs and chronic problems in the case of livestock, may not be reflected by elevated corticosteroid response. Consistent with earlier reports in goats [1,25,26], lymphocyte count decreased, and neutrophil count and N/L ratio increased with increasing transportation time. Increased glucocorticoids result in type 1/type 2 cytokine production, which in turn alters the balance of cellular and humoral immunity [27]. Habituation to trailer did not affect leukocyte counts in our study, since cortisol concentrations were also not influenced by TRT. However, Wickham et al. [17] observed that the N:L ratio due to transportation was significantly lower in transport-habituated sheep than in transport-naïve sheep, although our study did not involve habituation to transport.

Creatine kinase activity in blood is a good indicator of both muscle exertion and stress in goats [1,23]. In the skeletal muscle tissue, CK plays a vital role in energy production in both mitochondria and cytosol in two steps: (i) rephosphorylation of creatine in the mitochondria using ATP derived from oxidative phosphorylation, and (ii) regeneration of ATP using the phosphocreatine from the mitochondria [28]. The enzyme level in blood is also a measure of muscle damage or injuries, as CK seeps out of the muscle cells in response to physical exercise or stress due to an increase in permeability of sarcolemma [28], although the mechanism is not fully understood. Additionally, physical injuries, such as muscle fiber tears, may result in increased activities of blood CK. This is probably due to the release of the myofibrillar isoenzyme CK-MM from the M-line and I-band of sarcomeres where it meets the energy requirements under physiological conditions [29]. In the current study, CK activities in the H group tended to be lower than in the NH group. The CK activities noticed in both groups in the present study are lower than what we observed in a previous transportation experiment [1], but comparable to the values obtained in a recent study [19]. It is not clear if the H goats were less agitated by the handling and transportation processes and sustained fewer physical injuries than the NH goats since the difference between H and NH was not significant.

There was a differential effect of transportation time on CK levels in the H group compared to the NH group, resulting in a significant TRT × Time effect. Activity increased during the initial hours and toward the end of the transportation in the NH goats, which is a reflection of the handling and loading process and of transportation itself [30], as CK activity increases in blood about 2–3 h after encountering stress in goats [1]. Changes in CK activity over transportation time have been reported to occur due to temperature variation in the trailer, skeletal muscle activity to maintain posture in a moving vehicle, and/or feed deprivation [29,31,32]. In the present study, the CK activity was stable throughout transportation in the H group, while it appeared to be erratic in the NH group, probably due to intense muscle contractions. Evans et al. [33] reported that lower-intensity submaximal muscle voluntary contractions linearly corresponded with CK activities; however, higher-intensity muscle contractions altered the time course of CK release.

### 4.3. Catecholamines and Other Biogenic Amines

In our study, habituation did not have any significant effect on dopamine, epinephrine, and norepinephrine concentrations. Dopamine is produced in the substantia nigra and the ventral tegmental area in the midbrain, and the dopaminergic neurons extend to several other areas of the brain [34]. Since dopamine is present only in small amounts in the adrenal gland compared to epinephrine and norepinephrine, plasma dopamine is primarily from sympathetic noradrenergic nerves [35]. Its release, or metabolism changes in response to a stressor, are based on factors such as intensity and duration of stress. A novel stressor that is mild or moderate and short-lasting can activate dopamine release, while an intense, prolonged, and uncontrollable stressor has an inhibitory effect on dopamine release [36]. Our study supports the notion in that overall dopamine concentrations were higher during the initial stages, but generally decreased with transportation time. The dopamine concentrations in the H group were higher at preload, 0 h, and 2 h sampling, but decreased and stayed at a lower level throughout the rest of the journey. In the NH goats, however, the dopamine concentrations were greater during preload and 0 h sampling, and thereafter the levels appeared to fluctuate without a clear pattern. For instance, the mean concentration at 6 h was greater in the NH goats compared to the H goats, suggesting that the NH goats responded differently to extended transportation. The higher dopamine concentrations initially were likely due to the acute nature of the stress of handling, loading, and the onset of transportation itself. As the transportation stress was prolonged, dopamine reuptake and breakdown mechanisms were probably triggered, thereby decreasing the circulating concentrations. Changes in dopamine levels when animals are exposed to prolonged stress could also alter their behaviors [34]; however, the activities of goats were not monitored during transport in this study.

Plasma norepinephrine and epinephrine concentrations were also not influenced by the habituation treatment. In the present study, it is likely that epinephrine and norepinephrine increased due to the treatment, but quickly decreased due to uptake or breakdown by monoamine oxidase in neuronal cells (if not transported into storage vesicles) or by catechol-O-methyltransferase in non-neuronal cells [4].

Metanephrine and normetanephrine are O-methylated metabolites of epinephrine and normetanephrine, respectively. Although normetanephrine concentrations were not affected by TRT in the current experiment, metanephrine levels tended to be lower in the H goats compared to the NH goats, which indicates that the latter group likely experienced more stress during transportation. In addition, the Time effect for plasma metanephrine concentrations was significant, as the levels increased and peaked at 2 h in the NH group, while the levels increased rather gradually and moderately during the first 4 h of transportation in the H group before decreasing. The Time pattern noticed for metanephrine concentrations was similar to that for cortisol in this study, as both levels were higher during the initial hours of transportation and decreased thereafter. Batchu et al. [3] observed a significant correlation between cortisol and metanephrine concentrations in response to stress in goats and suggested that metanephrine could be a good indicator of stress under practical conditions. Increases in the blood concentrations of breakdown products of epinephrine and norepinephrine have been previously reported in rabbits in response to restraining stress [37].

Overall plasma phenylethylamine concentrations in the present study were higher in the H goats compared to the NH goats. β-phenylethylamine is a biogenic amine produced in the mammalian brain in trace amounts, primarily in the dopaminergic areas, by decarboxylation of phenylalanine [38]. It is also naturally present in some plant-based products, such as chocolate and wine [39,40]. Lapin [41] reported that mild doses of β -phenylethylamine in rodents increase dopamine levels, and depending on the extent to which it can increase dopamine concentrations, β -phenylethylamine is believed to increase locomotor activity similar to that of amphetamine [42]. However, dopamine concentrations were not affected by habituating goats to trailers in our study. Several authors have reported that elevated blood phenylethylamine concentrations can help animals in many ways. It has been reported to modulate physical energy, attention, and mood [6]. Since phenylethylamine can also partially control serotonin, dopamine, and norepinephrine, these neurochemicals can also influence mood [6]. In humans, patients with lower levels of phenylethylamine and breakdown metabolites in the blood suffer bouts of sadness [43]. These authors also speculated that phenylethylamine may be related to the curative effect of physical exercise on depression. Furthermore, it can combat the negative effects of corticosteroids in the body, as Lee et al. [44] observed that mice injected with corticosterone displayed depression-like behavior; however, phenylethylamine had an antidepressant effect in corticosterone-treated mice by modulating the BDNF/TrkB/CREB signaling pathway.

Plasma 5-methoxytryptamine concentrations were significantly affected by both habituation treatment and transportation time in the present study. The overall concentrations were higher in the H goats compared to the NH goats. Considered a neurohormone by some earlier investigators, 5-methoxytryptamine is formed by deacetylation of melatonin, decarboxylation of 5-methoxytryptophan, and methylation of serotonin [45,46,47]. Hara et al. [7] reported that 5-methoxytryptamine can confer antioxidant capacity during oxidative stress in rats, probably due to its indole structure and associated free-radical scavenging ability. Cannizzaro et al. [48] observed that in adolescent rats, perinatal exposure to 5-methoxytryptamine enhanced adaptive behavioral responses related to stress. Although 5-methoxytryptamine concentrations were higher in the H goats throughout the 10-h transportation period, the differences were significant at the 0-h and 8-h sampling periods. The higher concentrations of this biogenic amine in the H goats may indicate that habituation to trailers can be beneficial in providing better antioxidant capacity during prolonged stress in goats.

As most goat producers around the world are smallholder farmers, this simple and cost-effective method could be easily adopted by operations of any scale. With a little planning, farmers can implement this method to habituate goats to trailers for a few weeks before a scheduled long-distance/duration transportation of their goats. The method may not add to the cost, but can at the least make the loading process quicker and easier.

## 5. Conclusions

Habituation to trailers did not significantly reduce adrenocortical response in goats; however, it may facilitate the loading process. The fluctuating creatine kinase enzyme activities during transportation in non-habituated goats could also imply that these animals experienced intense muscle contractions. The pattern of dopamine concentrations over time in the treatment groups is further evidence that the non-habituated goats reacted differently during prolonged transportation compared to the habituated goats. Although energy stabilization and antioxidant status were not assessed in the current study, we speculate based on other reports that habituation to a trailer may improve these capacities in goats. Habituation to a trailer may improve mood and energy stabilization in goats during long-distance transportation, as evidenced by higher phenylethylamine concentrations in the habituated goats. The higher concentrations of this 5-methoxytryptamine also suggest that habituation can provide better antioxidant capacity during prolonged stress in goats. The effects of stress on blood phenylethylamine and 5-methoxytryptamine concentrations in goats and their practical value as indicators of stress need further investigation. Metanephrine concentrations may more accurately reflect stress levels in goats under practical conditions. Habituation of goats to livestock trailers prior to transportation that is inevitable in commercial operations may yield several benefits. This simple management method can be easily adopted by goat producers around the world.

## Figures and Tables

**Table 1:** Effects of habituation to livestock trailer on the behavior of Spanish goats after loading for transportation.

Behavior	Time ^1^								Treatment (TRT)				n ^6^	Time Effect ^7^	TRT Effect ^8^
	1st 5-min		2nd 5-min		3rd 5-min		4th 5-min		H ^4^		NH ^5^				
	Median ^2^	IQR ^3^	Median	IQR	Median	IQR	Median	IQR	Median	IQR	Median	IQR			
Standing	15	9	14	8	17	6	13	10	15	6.5	14.5	11.5	84	*p* < 0.01	NS ^9^
Moving	5	6	5	6	3	4	4.5	6	5	5.5	4	8.5	84	*p* < 0.01	NS
Agonistic	1	3	1	3	0	2	1	3.5	1	0	1	3	84	NS	NS
Climbing	0	0	0	1	0	0	0	0	0	0	0	1.5	84	NS	NS

^1^ 20-min period immediately after loading was divided into four 5-min segments, ^2^ Median number of goats performing the behavior. ^3^ Interquartile range (Q3-Q1, where Q1 = first quartile and Q3 = third quartile), ^4^ Habituated. ^5^ Non-Habituated, ^6^ Total of 4 replicates (21 goats/replicate/treatment group), ^7,8^ Time and TRT main effects by Friedman’s Two-Way ANOVA by Ranks Test (Non-parametric), ^9^ Not significant (*p* > 0.05).

**Table 2:** Effects of habituation to livestock trailer on neutrophil (N) and lymphocyte (L) counts and N/L ratio in Spanish goats.

Time	Neutrophil			Lymphocyte			N/L Ratio		
	TRT		Time	TRT		Time	TRT		Time
	H ^1^	NH ^2^		H	NH		H	NH	
Pre-Load	66.6	67.3	66.9 ^e^	32.3	36.5	34.4 ^a^	3.1	2.7	2.9 ^d^
0 h	72.8	74.7	73.7 ^cde^	25.9	24.1	25.0 ^b^	3.2	3.5	3.3 ^d^
2 h	80.6	80.7	80.8 ^abc^	18.2	23.1	20.6 ^bc^	5.2	4.9	5.1 ^c^
4 h	86.5	87.2	86.8 ^a^	12.7	12.1	12.4 ^c^	7.3	8.2	7.8 ^a^
6 h	82.8	78.2	80.5 ^abc^	16.4	20.9	18.6 ^bc^	6.0	6.8	6.4 ^ab^
8 h	81.3	86.3	83.8 ^abc^	17.7	12.9	15.3 ^c^	5.4	8.4	6.9 ^ab^
10 h	82.5	72.8	77.7 ^cd^	16.6	35.9	26.2 ^ab^	5.9	4.9	5.4 ^bc^
N ^3^	12	12	24	12	12	24	12	12	24
SEM	3.76	3.76	2.66	4.57	4.57	3.23	0.78	0.78	0.55
TRT ^4^	NS ^5^			NS			NS		
Time	*p* < 0.01			*p* < 0.01			*p* < 0.01		
TRT × Time	NS			NS			NS		

^a–e^ Time main effect means (average across TRT at each time point) with different superscripts differ significantly by LSD test at *p* < 0.05. ^1^ Habituated. ^2^ Non-Habituated. ^3^ Number of goats sampled per time period. ^4^ Treatment. ^5^ Not significant (*p* > 0.05).

## Data Availability

The data presented in this study are available on request from the corresponding author.
